# Efficacy and mechanism of sub-sensory sacral (optimised) neuromodulation in adults with faecal incontinence: study protocol for a randomised controlled trial

**DOI:** 10.1186/s13063-018-2689-1

**Published:** 2018-06-26

**Authors:** Eleanor McAlees, Paul F. Vollebregt, Natasha Stevens, Tom C. Dudding, Anton V. Emmanuel, Paul L. Furlong, Shaheen Hamdy, Richard L. Hooper, James F. X. Jones, Christine Norton, P. Ronan O’Connell, S. Mark Scott, Charles H. Knowles

**Affiliations:** 1 National Bowel Research Centre, Blizard Institute, Barts and the London School of Medicine and Dentistry, Queen Mary University of London, London, UK; 2 University Hospital Southampton NHS Foundation Trust, Southampton, UK; 3 University College Hospital, London, UK; 4 Aston Brain Centre, Aston University, Birmingham, UK; 5 University of Manchester and Salford Royal Hospital, Manchester and Salford, UK; 6 Pragmatic Clinical Trials Unit, Centre for Primary Care and Public Health, Queen Mary University of London, London, UK; 7 University College Dublin School of Medicine, Dublin, Ireland; 8 King’s College, London, UK; 9 National Bowel Research Centre, 1st Floor Abernethy Building, 2 Newark St, London, E1 2A UK

**Keywords:** Faecal incontinence, Sacral neuromodulation, Sacral nerve stimulation, Randomised controlled trial, Magneticoencepholograpy, Evoked potential, Anorectal manometry

## Abstract

**Background:**

Faecal incontinence (FI) is a substantial health problem with a prevalence of approximately 8% in community-dwelling populations. Sacral neuromodulation (SNM) is considered the first-line surgical treatment option in adults with FI in whom conservative therapies have failed. The clinical efficacy of SNM has never been rigorously determined in a trial setting and the underlying mechanism of action remains unclear.

**Methods/design:**

The design encompasses a multicentre, randomised, double-blind crossover trial and cohort follow-up study. Ninety participants will be randomised to one of two groups (SNM/SHAM or SHAM/SNM) in an allocation ratio of 1:1. The main inclusion criteria will be adults aged 18–75 years meeting Rome III and ICI definitions of FI, who have failed non-surgical treatments to the UK standard, who have a minimum of eight FI episodes in a 4-week screening period, and who are clinically suitable for SNM. The primary objective is to estimate the clinical efficacy of sub-sensory SNM vs. SHAM at 32 weeks based on the primary outcome of frequency of FI episodes using a 4-week paper diary, using mixed Poisson regression analysis on the intention-to-treat principle. The study is powered (0.9) to detect a 30% reduction in frequency of FI episodes between sub-sensory SNM and SHAM stimulation over a 32-week crossover period.

Secondary objectives include: measurement of established and new clinical outcomes after 1 year of therapy using new (2017 published) optimised therapy (with standardised SNM-lead placement); validation of new electronic outcome measures (events) and a device to record them, and identification of potential biological effects of SNM on underlying anorectal afferent neuronal pathophysiology (hypothesis: SNM leads to increased frequency of perceived transient anal sphincter relaxations; improved conscious sensation of defaecatory urge and cortical/subcortical changes in afferent responses to anorectal electrical stimulation (main techniques: high-resolution anorectal manometry and magnetoencephalography).

**Discussion:**

This trial will determine clinical effect size for sub-sensory chronic electrical stimulation of the sacral innervation. It will provide experimental evidence of modifiable afferent neurophysiology that may aid future patient selection as well as a basic understanding of the pathophysiology of FI.

**Trial registration:**

International Standard Randomised Controlled Trial Number: ISRCTN98760715. Registered on 15 September 2017.

**Electronic supplementary material:**

The online version of this article (10.1186/s13063-018-2689-1) contains supplementary material, which is available to authorized users.

## Background

Faecal incontinence (FI) is defined as the recurrent involuntary loss of faecal material leading to a social or hygienic problem (International Consultation on Incontinence: ICI) [1] and not related to an acute diarrhoeal illness (Rome III). While variations exist regarding prevalence due to differences in survey methods, screening questions, reference timeframe, definition and population studied, few could argue that FI is not a substantial health problem. Population studies suggest a prevalence ranging from 3 to 15% in community-dwelling women, 15% in community-dwelling older people, 18–33% in hospitals, 38% in home health, and up to 50–70% in nursing homes [2]. A clear relationship with advancing age suggests that it will remain a problem within the developing Western population demographic [2].

FI leads to substantive effects on quality of life in terms of physical and emotional health; to stigmatisation and social isolation; and in older people, admission to residential care. Societal costs incurred by lost work productivity and absenteeism can be added to significant direct and indirect medical costs attributable to drug and pad usage, to specialist care, and particularly to nursing costs in older patients. Such estimates probably under-reflect the full impact of FI due to under-reporting [3]. It is estimated that treatment of urinary and FI account for at least 2% of the total UK healthcare budget [4].

Initial treatments of FI include pharmacological and behavioural therapies, the latter generally incorporating some form of biofeedback. While anecdotally these treatments appear to improve continence in a significant number of patients there is little high-quality evidence to support this [5]. Traditionally, surgical treatments focussing on anal sphincter function are offered when conservative measures fail. These can be classified into reconstructive (sphincteroplasty), augmentation (bulking agents) and neosphincter procedures (artificial sphincters, graciloplasty). These procedures are invasive, irreversible, and balance variable success rates against some risk of significant morbidity. A stoma is the final option.

Neuromodulation is one of the fastest growing areas of medicine: technologies now address diverse disease areas including epilepsy, Parkinson’s disease and tremor, chronic pain and deafness. The application of neuromodulation to the problem of FI has significantly changed the treatment paradigm for many patients over the past 20 years. Chronic stimulation of the sacral nerve roots using an implanted electrode and generator – sacral neuromodulation (SNM) is now considered the first-line surgical treatment option for the majority of adults with FI in whom non-operative therapies have failed to alleviate symptoms (NICE 2007 [4]) especially as it is the least invasive procedure. However, despite having regulatory approval from the NICE and the U.S. Food and Drug Administration (FDA), SNM remains an expensive intervention with some limitations in terms of a high-quality evidence base for either mechanism of action or efficacy.

### Evidence of SNM efficacy

Numerous observational studies show that SNM leads to substantial health gain for adults with FI with low levels of operative morbidity compared to alternative surgical strategies [6]. Reduced FI episodes correlate with objective QoL improvements [7] and SNM has been shown to be cost-effective with an ICER of £25,070 per quality-adjusted lif e year (QALY) lying within the threshold recommended by NICE as an effective use of NHS resources [7]. This systematic review, however, also highlighted the generally poor methodological quality of included studies which were almost universally single-centre retrospective or prospective clinical case series with unblinded observers and failure to report outcomes on an intention-to-treat (ITT) basis. The latter point is especially important since significant attrition bias undermines nearly all studies even including the higher-quality pivotal trial for FDA approval (a prospective multicentre US case series of 120 patients [8, 9]). Two independent publications from Europe that have reported large patient series using the ITT principle have shown less encouraging results (circa 45% long-term success) [10, 11].

Available randomised trial data for SNM in FI have recently been systematically reviewed [12]. A total of six included studies comprised four randomised crossover designs and two parallel-group randomised controlled trials (RCTs). One crossover included only two patients [13]; a further study published only in abstract form reported mainly mechanistic outcomes in only seven patients [14]. The remaining two crossover studies included the widely cited study by Leroi et al. [15], which enrolled 34 patients pre-selected on the basis of a successful prior SNM implantation. Only 27 participated in the crossover and only 24 completed the study (10 excluded patients included four explantations due to AEs and others due to lack of efficacy or protocol violations). Although the majority (18 / 24) of analysed patients preferred ‘ON’ vs. ‘OFF’ at the end of study, the study failed to show a clinically meaningful reduction of symptoms between ON and OFF periods; e.g. difference in median FI episodes per week of only one episode. This was suggested to result in part from a short washout period (1 week) and a carry-over effect. A second published crossover study [16] employed an identical trial design but with smaller numbers of patients, randomising only 16 of 31 preselected implanted patients and thence only for two 3-week crossover periods. In contrast to the earlier study, significant decreases in FI episodes and summative symptom scores were observed in the ON vs. OFF periods despite having no washout. In an unblinded RCT by Tjandra et al. [17] 53 participants with severe faecal incontinence in the SNM group experienced fewer episodes of faecal incontinence compared to the control group which received optimal medical therapy (mean − 5.20, 95% CI − 9.15 to − 1.25 at 3 months; mean − 6.30, 95% CI − 10.34 to − 2.26 at 12 months). Finally, an observer-blinded RCT of SNM vs. a less invasive form of neuromodulation: percutaneous tibial nerve stimulation (PTNS) [18] demonstrated a within-group effect size that was greater for SNM than PTNS. While pilot in design and with small numbers (*n* = 40 total), this effect was modest compared to most observational case series.

### Evidence of SNM mechanism

Traditional understanding of the pathophysiology and surgical management of FI held that the sphincter ‘barrier’ had primacy. It is now clear that while sphincter disruption is still relevant to the development of FI in many patients; e.g. obstetric injuries, it is only one factor in complex defaecatory dysfunction that involves alteration in unconscious anorectal and pelvic reflexes and conscious modulation by the central nervous system (CNS). SNM was developed for FI with the view that it would augment defective sphincteric function [19]. It is now well appreciated that patients with FI resulting from pathophysiology other than primary sphincter dysfunction also benefit from treatment [20]. The importance of sensory dysfunction on both urinary and bowel control is being increasingly appreciated and there is strong evolving evidence (including our own pilot data in humans and experimental animals) that the mechanism of action of SNM results primarily from modulation of afferent nerve activity.

Pulling the above evidence together it is clear that the clinical efficacy of SNM has never been rigorously determined in a trial setting. There is, therefore, a need for a well-designed study of SNM that seeks to determine definitive proof of clinical effect size and which notably improves on the small number of existing randomised studies and observational data. Such a study has the opportunity to embed a hypothesis-led mechanistic study.

## Methods/design

### Overall study aim

To determine clinical efficacy of sub-sensory, chronic, low-voltage, electrical, sacral nerve-root stimulation (SNM), using a commercially-available implantable device, Medtronic Interstim® in adults with FI failing conservative treatment.

### Objectives

#### Primary clinical objectives

1. To determine whether chronic, sub-sensory SNM leads to a minimum clinically-relevant reduction in frequency of total FI episodes compared to SHAM stimulation
  Hypothesis: SNM reduces frequency of total FI episodes by a mean of 30% compared to SHAM stimulation in the third month of chronic stimulation
2. To determine the effect size of sub-sensory SNM on a range of clinical outcomes compared to SHAM stimulation
  Hypothesis: sub-sensory SNM leads to significant and clinically-beneficial changes in a range of established and novel innovative outcome measures in the third month of chronic stimulation

#### Secondary clinical objectives

1. To provide 12-month clinical outcome data for SNM using optimised therapy (standardised lead placement): cohort follow-up study
2. To validate new electronically recorded outcome measures for future FI trials (and a new device to record them)
3. To provide data on the kinetics of response and carryover effects
4. To provide data on predictive value of baseline characteristics and operative factors as covariates of response (especially on optimised lead placement)
5. To increase general understanding of the basic pathophysiology of FI in a well-characterised patient cohort

#### Mechanistic objectives

1. To determine the effect of sub-sensory SNM on anorectal sensorimotor reflex function
  Hypotheses: (1) SNM but not SHAM increases frequencies of fasting and fed perceived and unperceived transient anal sphincter relaxations (TASR) (based on prolonged high-resolution anorectal manometry recordings) to levels observed in healthy individuals; (2) SNM but not SHAM increases conscious sensation of defaecatory urge based on symptom reporting and objective measures of anorectal sensory function
2. To determine the effect of SNM on anocortical afferent function
  Hypothesis: SNM leads to brain plasticity (based on magnetoencephalography (MEG)) in motor and non-motor cortical and sub-cortical regions

### Eligibility criteria

#### Inclusion criteria

- Adults aged 18–75 years
- Meet Rome III and ICI definitions of FI (recurrent involuntary loss of faecal material that is a social or hygienic problem and not a consequence of an acute diarrhoeal illness)
- Failure of non-surgical treatments to meet the NICE standard*
- Minimum severity criteria of eight FI episodes in a 4-week screening period (this is important to exclude patients who might thence have zero FI episodes during baseline evaluations)
- Ability to understand written and spoken English or relevant language in European centres (due to questionnaire validity)
- Ability and willingness to give informed consent

* Minimum NICE standard includes; diet, bowel habit and toilet access being addressed. Medication, e.g. loperamide, advice on incontinence products, pelvic-floor muscle training, biofeedback and rectal irrigation should be offered if appropriate [4].

All patients will have been determined as clinically suitable for SNM based on clinical evaluation and subsequent multidisciplinary team discussion (as mandated by NHS England specialist commissioning guidance) or equivalent guidance in other participating EU countries.

#### Exclusion criteria

A standard list of exclusions (disease variants; surgical fitness, specific contraindications to implantation) will be used. Note that these are routine clinical exclusions to the use of SNM rather than participation in the research. For completion:

- Known communication between the anal and vaginal tracts
- Prior diagnosis of congenital anorectal malformations
- Previous rectal surgery (rectopexy / resection) performed < 12 months ago (24 months for cancer)
- Present evidence of full-thickness rectal prolapse
- Prior diagnosis of chronic inflammatory bowel diseases
- Symptoms of chronic constipation with over-flow incontinence
- Structural abnormality of the pelvic floor leading to clear evidence of obstructed defaecation based on examination and/or imaging
- Symptoms of significant evacuatory dysfunction based on Obstructive Defecation Syndrome Score ≥ 8
- Presence of active perianal sepsis (including pilonidal sinus)
- Defunctioning loop or end stoma in situ
- Diagnosed with neurological diseases, such as diabetic neuropathy, multiple sclerosis and Parkinson’s disease
- Current or future need for MR imaging based on clinical history
- Complete or partial spinal cord injury
- Bleeding disorders, e.g. haemophilia, warfarin therapy
- Pregnancy or intention to become pregnant during the study period
- Not fit for preferred method of anaesthesia
- Anatomical limitations that would prevent successful placement of an electrode including congenital abnormalities
- Psychiatric or physical inability to comply with the study protocol (including e-diary assessments) at the investigator’s discretion
- Is required to drive for long periods of time, e.g. lorry drivers, taxi drivers and delivery drivers

### Trial design

The overall design encompasses a randomised, double-blind crossover trial and a follow-up cohort study. The trial will be conducted in about 20 European centres (UK and Germany) and in Ireland.

#### Randomised, double-blind crossover design overview

**Fig. 1:**
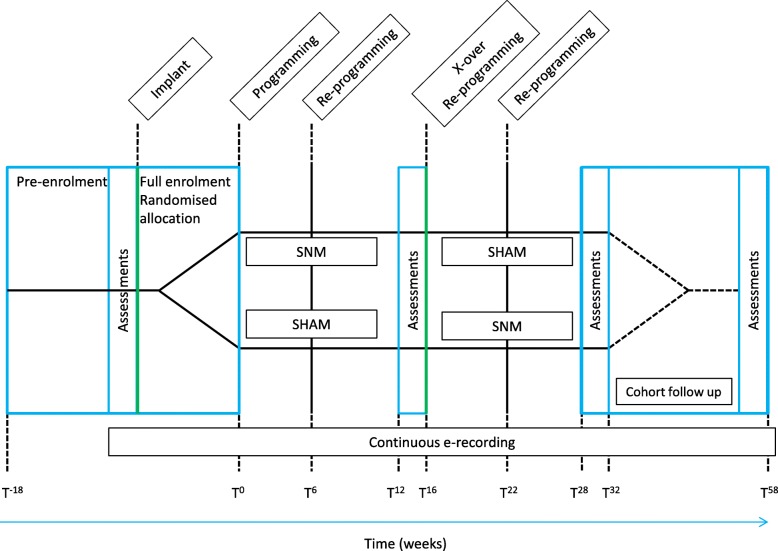
Flow diagram

**Fig. 2:**
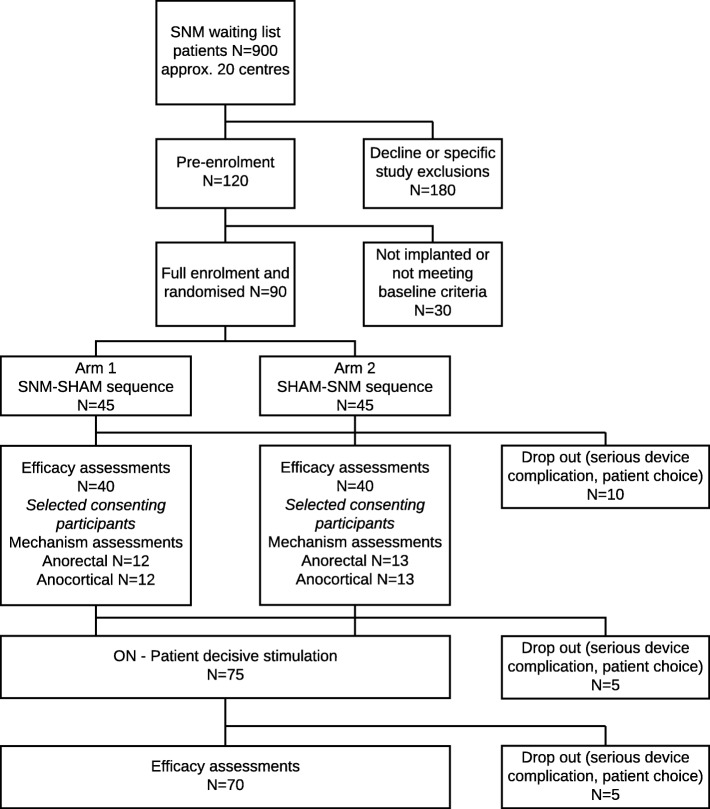
Study scheme diagram

Ninety eligible participants will be randomly allocated to two study arms after SNM implantation (see flow diagram below (Fig. 1) and study scheme diagram (Fig. 2)). Both arms have two intervention periods of 16 weeks’ duration (T0–T16 and T16–T32). Efficacy outcomes are derived from assessments in the final 4 weeks of each crossover period (T12–16 and T28–32), thus allowing for almost 3 months’ intervention before outcome assessments. A reprogramming session will be conducted by the routine clinical care team at 6 weeks in both periods of both arms (T6, T22). Time points will have an interval tolerance of ± 1 week for logistical expedience.

Mechanism studies will be performed in a subgroup of consecutively consenting patients equally from both arms (to avoid risk of performance bias) until saturation (*n* = minimum 20; aim 25 for both anorectal and anocortical studies) in the final 2 weeks of the 4-week assessment periods.

#### Cohort study: 12-month outcomes

After completing the crossover section of the study, patients will continue to be followed up for a further 26 weeks (estimated *n* = 75: allowing for dropouts). During this time they will have ‘open-label’ patient-decisive stimulation (sub- or supra-sensory) as would be normal for routine clinical practice. Further efficacy outcomes will be recorded at T54–58. While it is accepted that these do not represent true 1-year outcomes (16 weeks has been SHAM treatment during the crossover), these will give an indication of the short-term effectiveness of SNM using the optimised lead placement and within the rigor of a CTU-monitored randomised prospective study.

### Study procedures

#### Recruitment and consent procedures

Patients will be consecutively assessed for broad eligibility (using the inclusion/exclusion criteria checklist) from the surgery waiting lists of participating centres and counselled in detail about the study prior to any surgery; i.e. before test stimulation (pre-enrolment). A minimum of 24 h will be provided to enable consideration of patient information sheets (PIS) and the study requirements. Consent for screening and future enrolment will be conducted face to face in a private setting with an appropriately trained and delegated member of the clinical or research team. Patients will consent to the study (T–18: see Fig. 1) up to 4 weeks prior to surgery.

#### Randomisation procedures

- Group 1 (45): SNM/SHAM
- Group 2 (45): SHAM/SNM

Randomised allocation (1:1) will be performed at the time of surgery using a computer-based programme developed by the PCTU and stratified by sex and centre with block sizes of 4. The inclusion of sex as a stratification factor is justified by the potential differences in pathophysiology in the small number of male patients with significant FI [21]. Patients will be randomised prior to surgery so they enter the study even if it is not possible to implant the stimulator. If there is any problem with the online randomisation system, randomisation can be delayed up until the initial programming giving a window of 2 weeks; alternatively, emergency randomisation may be performed by an unblinded member of the coordinating team.

#### Blinding procedures

Research investigators and participants will be blinded to intervention status (SNM or SHAM). Patients will be informed of the allocation ratio of 1:1 and that blinding prevents them from knowing in which group they are participating (and, therefore, their order of intervention sequence). Patients will be issued with a patient programmer (InterStim iCon Patient Programmer Model 3037) with tamper-proof tape cut so as to obscure the stimulator setting but not obscure the on-off icon (which is in the top left-hand corner of the screen). This enables the patient to switch off the stimulator in an emergency; e.g. unwanted neurological AEs (the only emergency that would require this) and to permit driving (manufacturer’s guidance recommends that the stimulator should be turned off for driving). When the patient has completed their car journey they will simply reactivate the device which will return to the pre-set level (SNM or SHAM). This is a pragmatic consideration that is both necessary to complete the study (recruitment would be impossible if patients could not drive for the whole 32-week crossover period) and part of ‘real-life’. There is published evidence that switching the device off for part of the day (even for long periods) has no effect on efficacy over a chronic stimulation period [22–24]. The settings on the device (to turn stimulation settings up or down) will not be accessible to the participants, having been disabled at the time of programming, in addition to the external buttons being covered with tamper-proof tape. The patient programmer power switch, neurostimulator synchronisation switch and neurostimulator on/off switch will be accessible to the patient.

The Model 8840 Clinician programmer is able to access log data of stimulation usage so there is potential to check all data on ON-OFF cycling during the study intervention periods if this is required to validate fidelity of the intervention (a bit like used blister-packs to count unused drugs in a drug trial). During the SHAM period the neurostimulator will be active but not be providing stimulation (current set to 0 V). Therefore, analysis of neurostimulator activity in the SNM and SHAM phases should be equivocal in percentage of neurostimulator ‘use’ and un-blinding one participant would not compromise blinding for the remainder. The digital programming unit (N’Vision Clinician Programmer Model 8840) will not be supplied to the patient but can be used post hoc to determine if the patient has changed settings or switched stimulation on or off during the study (the programmed settings will also have been recorded on a case report form (CRF) by the unblinded clinical team member). The patient will not be removed from the study if the tamper-proof tape has been broken. This will be recorded for statistical analysis.

A nominated member of the research team or normal care clinician will have access to the programmer at the relevant fixed time points for stimulator adjustment (crossover and 6-week reprogramming). This person who will not be blind to intervention status will not otherwise be involved in the research protocol; e.g. outcomes assessments, collection of CRFs, data management.

### Planned interventions

#### Sacral neuromodulation (SNM) (Medtronic Interstim®)

The intervention is chronic low-voltage stimulation of the third sacral root using surgical implantation of a commercially available CE-marked active implantable (class III) medical device (Medtronic Interstim®) used in accord with the manufacturer’s instructions.

Patients meeting the mandated response using the monopolar temporary wire or quadripolar tined lead (lead choice and duration of testing based on local surgical practice) will undergo implantation of the permanent InterStim system under general or local anaesthesia (with sedation) by trained expert colorectal surgeons following the procedural steps developed by Siegel [25] and now published as full guidance [26] (in brief: fluoroscopic-aided percutaneous insertion of 3889 lead using curved stylet and accepting position only when three out of four electrodes provide low-voltage (< 3 V) contraction of the anal sphincter and pelvic floor ± big toe). The implantable pulse generator (3058; Medtronic) will be placed as pre-marked in the ipsilateral buttock only if electrode responses meet the Siegel criteria.

The device will be activated as per local policy. This can be in the post-operative period the same day as surgery or after a surgical stabilisation period of up to 2 weeks (this is routine clinical practice in some centres).

General programming parameters will accord with a written algorithm based on best clinical practice. Prior to programming, an impedance check will be performed and recorded to ensure integrity of the electrical system. The clinical team will set the electrode configuration to achieve sensory threshold defined as the stimulation amplitude where the patient feels the first sensation of stimulation in the anus or perineum (or vagina) at a 14-Hz frequency, pulse width 210 μsec (a perception of anal sphincter stimulation is considered by most to be optimal). To determine the amplitude necessary to elicit an anal sensation, the amplitude will be increased by 0.1 V from zero until the sensory threshold is reached [27]. The dominant electrode will be defined by initial mono-polar testing of each electrode noting the site of sensation and sensory threshold with each electrode used. The optimal electrode configuration will then be determined based on the programming algorithm. The amplitude required to elicit the sensory threshold with the optimal electrode configuration will be recorded.

The patient will continue with stimulation at sensory threshold for 5 min, and the process then repeated to identify the habituated sensory threshold. Sub-sensory chronic stimulation will then be performed at the level of the habituated sensory threshold [15] setting the device at this level. The maximum stimulation setting will be set at the habituated sensory threshold to ensure that an individual patient is unable to increase the amplitude of stimulation to above the sensory threshold and, therefore, determine whether they are receiving active stimulation or not.

At the 6-week time point after device activation, the patient will be re-assessed by the un-blinded research delegate or clinician. Changes in electrode configuration will be permitted if a patient is having sub-optimal efficacy or significant unwanted effects of stimulation. Any change in electrode configuration or site of sensation will be documented. The habituated sensory threshold will be re-calculated and stimulation thence returned to this level.

#### SHAM stimulation

Device implantation and post-operative optimisation proceeds as above. The habituated sensory threshold is recorded identically. However, the device is then returned to 0 V and (device remains on but will provide no stimulation). At the 6-week time point after device implantation, the patient is re-assessed for sub-optimal efficacy (anticipated in the majority if the fundamental hypothesis is correct) by the un-blinded research delegate or clinician. To maintain blinding, an identical procedure is followed as above; i.e. re-evaluation of sensory threshold and electrode configurations but this is followed by returning the stimulator to 0 V.

#### Procedures for mechanistic studies (subgroup of patients)

Because mechanistic studies involve quite burdensome studies and because anocortical (MEG) studies can only be performed at the Wellcome Trust Laboratory for MEG studies, Aston Brain Centre by highly experienced investigators (Furlong, Hamdy), two separate cohorts of patients will be recruited and separately consented for anorectal and anocortical studies. The numbers of patients for each will be defined by ability to recruit and retain patients in these studies and are in part a function of geographical location of recruitment; however, we will aim to recruit 25 patients to the both anorectal and anocortical studies (see ‘Sample size’ section).

##### Anorectal studies

Patients in the London area (several centres) will be identified as potential subjects and provided with the specific PIS. Interested patients will need to make two visits to the GI Physiology Unit at Barts Health NHS Trust. Patients will undergo quick (clinically routine) tests of anal and rectal sensory function. The high resolution manometry catheter (Medical Measurement Systems) is then inserted and a standard (clinically routine and internationally agreed) protocol [28] of basic pressure measurements obtained. Thereafter, the patient will undergo a prolonged recording (total 1.5 h) of anorectal pressures at rest in a semi-recumbent position in a private room before and after a test meal (45 min each phase). During this time, they can watch TV but will be instructed to press an event recorder for any episodes of ‘urge’ or passage of flatus and complete a sensation record. The catheter is then removed and the study is finished.

#### Anocortical studies

Patients in the Midlands area (Sandwell and West Birmingham NHS, University Hospital Birmingham, Heart of England NHS and University of Leicester NHS Trusts) will be identified as potential subjects and provided with the specific PIS. Interested patients will need to make a total of three visits to the Aston Brain Centre.

**Fig. 3:**
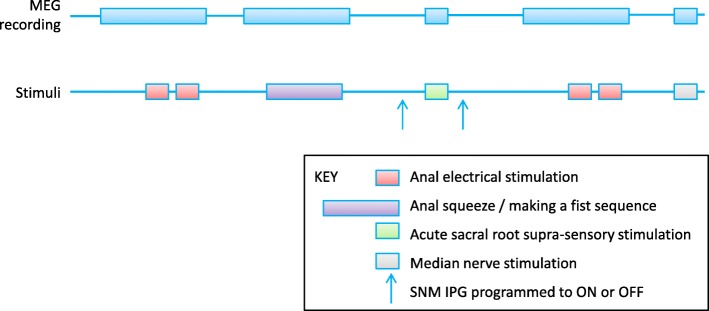
Magnetoencephalography (MEG) protocol for anocortical study

Only patients known to be proceeding to implantation will be invited for baseline evaluation and this can only proceed after removal of the test electrode (due to magnetic resonance imaging (MRI)) or in those in whom there is good certainty that a tined lead evaluation will progress to implantation. A baseline MEG will be acquired according to the specific protocol developed and tested by the applicants (see Fig. 3). At the same visit (but after the MEG) they will have an MRI head scan. At the second and third visits (SNM or SHAM in random sequence), the patient will have further MEG acquisitions only.

### Study visits

**Fig. 4:**
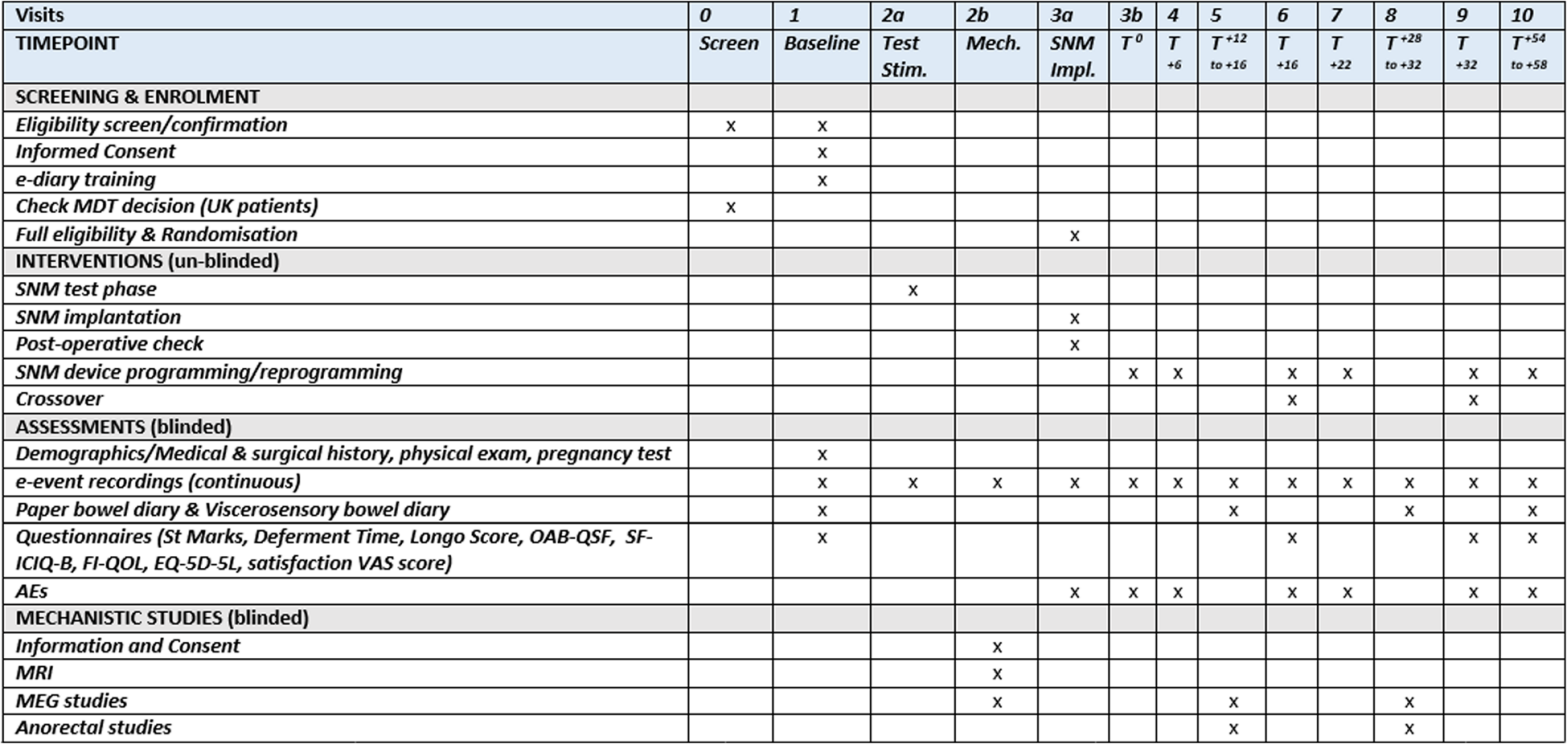
Standard Protocol Items: Recommendations for Interventional Trials (SPIRIT) Figure

The study visits are shown in the Standard Protocol Items: Recommendations for Interventional Trials (SPIRIT) Figure (Fig. 4; Additional file 1).

#### Visit 0: Screening

Prior to visit 1 patients will be initially assessed for eligibility against the inclusion and exclusion criteria checklist. The multidisciplinary pelvic floor multidisciplinary team (MDT) discussion needs to be reviewed prior to visit 1. Eligible patients will be sent the REC-approved invitation letter and PIS and invited to attend the visit 1 baseline visit. All patients screened will be added to the screening log.

All study visits have a window of ± 1 week for logistical purposes.

### Screening and baseline visits

#### Visit 0: Screening

Prior to visit 1 patients will be initially assessed for eligibility against the inclusion and exclusion criteria checklist. Pelvic floor MDT discussion needs to be reviewed prior to visit 1. Eligible patients will be sent the REC-approved invitation letter and PIS and invited to attend the visit 1 baseline visit. All patients screened will be added to the screening log.

#### Visit 1: Baseline

Eligibility against the inclusion/exclusion criteria will be reviewed, then, after discussing the study and PIS, patients in agreement complete written informed consent. This visit must take place no more than 18 weeks before permanent implantation.

Once a patient has been consented they will have the following assessments:

- Demographics, standardised medical/surgical history taken including history of incontinence symptoms, gynaecological history and pregnancy test (women of childbearing potential)
- Clinical examination of perineum, anus and rectum (if not documented previously within 6 months)
- Baseline outcome assessments: St Mark’s continence score, Deferment time, Longo Score, OAB-Q Short Form, International Consultation on Incontinence Bowel (SF-ICIQ-B) questionnaire, Faecal Incontinence Quality of Life (FI QoL) score and EQ-5D-5 L/Visual Analogue Scale (VAS)

At this visit patients will also be given the 4-week paper bowel diary (which will also record loperamide usage and taught how to use the touch-screen electronic device), which will be started from this visit.

A total of 4 weeks is provided to complete the diary. A viscerosensory bowel diary will also be provided with instructions for completion over 5 days.

### Surgical intervention visits

#### Visit 2a: Test stimulation

A 4-week window must be given between baseline and test stimulation to allow for the completion of the baseline bowel diary. Test stimulation will take place according to routine care, this will require the patient to attend the hospital as an outpatient, and no research data collection is required. Test stimulation is, therefore, not considered a study intervention and will be performed in accord with local clinical practice. Based on previous data [6, 18], 15% of patients will fail temporary SNM evaluation and will not proceed to permanent implantation.

#### Visit 2b: Mechanistic study enrolment

Before permanent device implantation, those participants passing the test stimulation phase or those patients to have tined lead insertion with a high probability of going through to permanent stimulation, will be selected for and consented to the mechanistic study. All patients must have completed the 4-week bowel diary.

Those selected for anocortical studies will then receive the following investigations:

- MRI head
- MEG to electrical anal stimulation, anal squeeze, sacral-root suprasensory stimulation, median nerve stimulation

#### Visit 3a: Permanent device implantation (SNM implant)

Following test stimulation patients will be admitted as a day case for permanent device implantation. Eligibility for randomisation will be re-confirmed (assessment baseline diary data). This visit must occur no later than 18 weeks after the baseline visit.

Patients are randomised prior to knife to skin to either one of the two groups:

- Group 1 will the initially receive sacral neuromodulation and
- Group 2 will initially receive SHAM stimulation

Sixteen-week SNM or SHAM periods will be counted from the initial programming not from the day of surgery.

Intraoperative data will be collected including:

- Lead position – radiological side and foramen level. Number of electrodes in foramina
- Motor thresholds for each of the four electrodes on the quadripolar lead
- Physiological motor (± sensory) response for the chosen foramen for lead implantation
- Other intraoperative data: length of op, type of anaesthesia (including use of any paralyzing agent), blood loss, any other complications

#### Visit 3b: Initial programming (T0)

Post-operatively the implant will undergo baseline checks using impedance measurements of the four electrodes to ensure integrity of the electrical system. Patients will have their SNM programmed as per routine care. This can be done in the post-operative recovery period or up to 2 weeks post surgery. All further follow-up visits will be counted from the initial programming not from the day of surgery.

To reduce selection bias, no consenting patient with an implant in situ will be excluded from participation; i.e. regardless of the surgeon’s views on success or otherwise of implantation. At each follow-up visit impedance measurements will be repeated to ensure maintained integrity of the electrical system. If a closed or open circuit is detected (suggesting possible neurostimulator or lead malfunction) then this will be documented. If satisfactory sensory response can be achieved using an alternative electrode configuration then the patient will be reprogrammed and can continue in the study. In the absence of a satisfactory sensory response with an abnormal impedance measurement the patient will still be followed up as per ITT and any changes to treatment will be recorded in the deviation log.

At each visit any change in electrode configuration, sensory threshold and location of maximum bodily sensation will be recorded. The percentage of time the implant has been active for will be recorded and the usage counters reset.

All programming will be performed using the Model 8840 N’Vision clinical programmer. The patient programmer can, therefore, be covered with tamper-proof tape for the entire clinical trial and no access is required to this device apart from to the power on/off button, synchronisation button and implant on/off button.

Following initial programming:

- Group 1: the sub-sensory amplitude will be recorded along with the electrode configuration used.
- Group 2: the sub-sensory amplitude will be recorded along with the electrode configuration used before returning the amplitude to 0 V

Any AEs will be collected at this visit and all subsequent face-to-face visits.

### Crossover phases T0 to T32

#### Visit 4: 6-week reprogramming visit (T + 6)

The tamper-proof tape is left on the patient’s programmer, programming is done via the clinician’s programmer.

- Group 1: patient assessed for sub-optimal efficacy or unwanted effects of stimulation. In the presence of sub-optimal efficacy or adverse effects the electrode configuration can be changed as per reprogramming algorithm. The sensory threshold is once again recorded and device returned to the sub-sensory setting
- Group 2: the sensory threshold is recorded and the electrode configuration can be changed if the site of stimulation appears to be sub-optimal (aim for anal stimulation) before returning device to 0 V

#### Visit 5: Assessment (T + 12 to + 16)

All patients will start the 4-week paper bowel diary and 5-day viscerosensory diary. This can be sent by mail or email, a face-to-face visit is not required.

The selected subgroup will have the first of the mechanistic follow-up studies completed (MEG or Anorectal).

#### Visit 6: Crossover visit (T + 16)

At crossover, the device is turned off for 20 min followed by re-evaluation of the sensory threshold and best electrode configuration in the manner outlined above. The intervention is then reversed for each arm.

Paper diary is completed and returned. Follow-up assessment questionnaires (St Mark’s continence score, Deferment time, OAB-Q Short Form, International Consultation on Incontinence Bowel (SF-ICIQ-B) questionnaire, FI QoL score and EQ-5D-5 L/VAS). Patients will also record their satisfaction on a Likert scale.

#### Visit 7: 6-week reprogramming visit (T + 22)

All patients will have a further follow-up 6 weeks after crossover at T22.

Leaving the tamper-proof tape on the patient’s programmer, programming is performed using the clinician’s programmer.

- Group 1: the sensory threshold is recorded and the electrode configuration can be changed if the site of stimulation appears to be sub-optimal (aim for anal stimulation) before returning device to 0 V
- Group 2: patient assessed for sub-optimal efficacy or unwanted effects of stimulation. In the presence of sub-optimal efficacy or adverse effects the electrode configuration can be changed as per reprogramming algorithm. The sensory threshold is once again recorded and the device returned to the sub-sensory setting

#### Visit 8: Assessments (T + 28 to + 32)

All patients will start the 4-week paper bowel diary and 5-day viscerosensory diary. This can be sent by mail or email, a face-to-face visit is not required.

The selected subgroup will have the second of the mechanistic follow-up studies completed, (MEG or Anorectal).

### Open-label cohort follow-up T32–58

#### Visit 9: End of crossover (T + 32)

At 32 weeks (and after collection of final crossover study data), patients will enter the follow-up phase with patient-decisive stimulation (sub- or supra-sensory) as would be normal for routine clinical practice. A member of the clinical team will reprogramme the device accordingly. Further programming and advice can be provided as per routine care during the period 32–58 weeks. All visits or contact with the clinical team during this time will be recorded on the Note to File CRF.

The 4-week paper bowel diary and 5-day viscerosensory diary will be completed and returned at this visit and the set of follow-up assessment questionnaires (St Mark’s continence score, Deferment time, OAB-Q Short Form, International Consultation on Incontinence Bowel (SF-ICIQ-B) questionnaire, FI QoL score and the EuroQol Health Outcome Measure (EQ-5D-5 L)/VAS). Patients will also record their satisfaction on a Likert scale).

#### Visit 10: Final assessment (T + 54 to + 58)

Patients will be asked to complete a further paper bowel diary and 5-day viscerosensory diary for the last 4 weeks (T54–58). During the final visit both the e-diary and paper diaries will be collected. Patients will undergo final reprogramming and complete the outcome questionnaires and Likert scale. Any AEs will be reviewed and resolved. Patients will then be discharged from the study and continue with normal clinical care.

### Concomitant care and interventions

It is inevitable that participants will seek recourse to loperamide and other medications during the course of the programme. Breakthrough loperamide usage is captured on the patient diary and St Mark’s continence questionnaire (see ‘Outcomes’ section). Additional concomitant medication reporting is not required for assessment of eligibility or safety monitoring; e.g. contraindication with the intervention. Thus, concomitant medications will not be recorded.

### Discontinuation criteria (participants and study)

Clinical care will take priority. The intervention plan allows the direct care team to remain autonomous in clinical decisions and modify their approach accordingly. It is unlikely that the intervention will need to be formally discontinued. However, if the direct care team or the research team at any point feel that the intervention is affecting the patient’s recovery, outcome or prognosis then it will be discontinued immediately. The events and circumstances will be recorded. If any safety concerns have arisen, these will be reported according to research governance framework guidelines.

### Withdrawal criteria

Patients can withdraw at any point in the study. The data collected from consent to the point of withdrawal will be kept for the ITT analysis, as outlined in the patient information and consent form.

Patients will be withdrawn from treatment but follow-up data will be continued to be collected if they:

- Electively withdraw from treatment
- Are not fit for surgery
- Become pregnant or intend to become pregnant
- Are unable to participate due to an concurrent severe illness
- Develop an acute psychological illness causing concerns

Patients will be withdrawn from both treatment and follow-up if they:

- Choose to withdraw from treatment and follow-up data collection
- Become lost to follow-up (after at least three attempts at contact by research/clinical staff using at least two different methods)
- Die or become severely incapacitated so follow-up data collection is impossible

### Criteria for early termination

If the DMEC, TSC, REC or sponsor determine it is within the best interests of the participants or trial to terminate the study, written notification will be given to the CI. This may be due to, but not limited to; serious safety concerns, success or failure of the primary outcome, serious breaches, acts of fraud, critical findings or persistent non-compliance that negatively affects patient safety or data integrity. If the study is terminated participants will be returned to the normal follow-up and routine care.

### Outcomes

#### Primary clinical outcome

The reduction in FI events in SNM vs. SHAM phase of crossover (16 and 32 weeks).

Frequency of FI episodes per unit time will be patient-recorded using 4-week paper bowel diaries. While the limitations of this method are well established [29], this remains the ‘gold standard’ in FI [15, 18, 27, 30] (we will, however, be recording for 4 weeks rather than only 2 as in many previous studies). The measure of treatment effect is the average number of FI events per 4-week period for patients undergoing SNM as compared with the average number of events for patients undergoing SHAM simulation. The study is powered to detect a ratio of 0.7. This is not to be confused with the reduction in the actual number of events post intervention for a given patient, where a 50% reduction has frequently been employed, albeit subjectively, to define ‘success’ for that patient [18, 30]. Rather, we use number of events as a quantitative outcome, achieving greater power than a dichotomous outcome of successful/unsuccessful, and we power to detect a 30% reduction, on average, in this outcome on ITT principles.

The paper diary will be completed prior to implantation then at the end of each crossover phase and again at the end of the cohort follow-up.

#### Secondary clinical outcomes

**Fig. 5:**
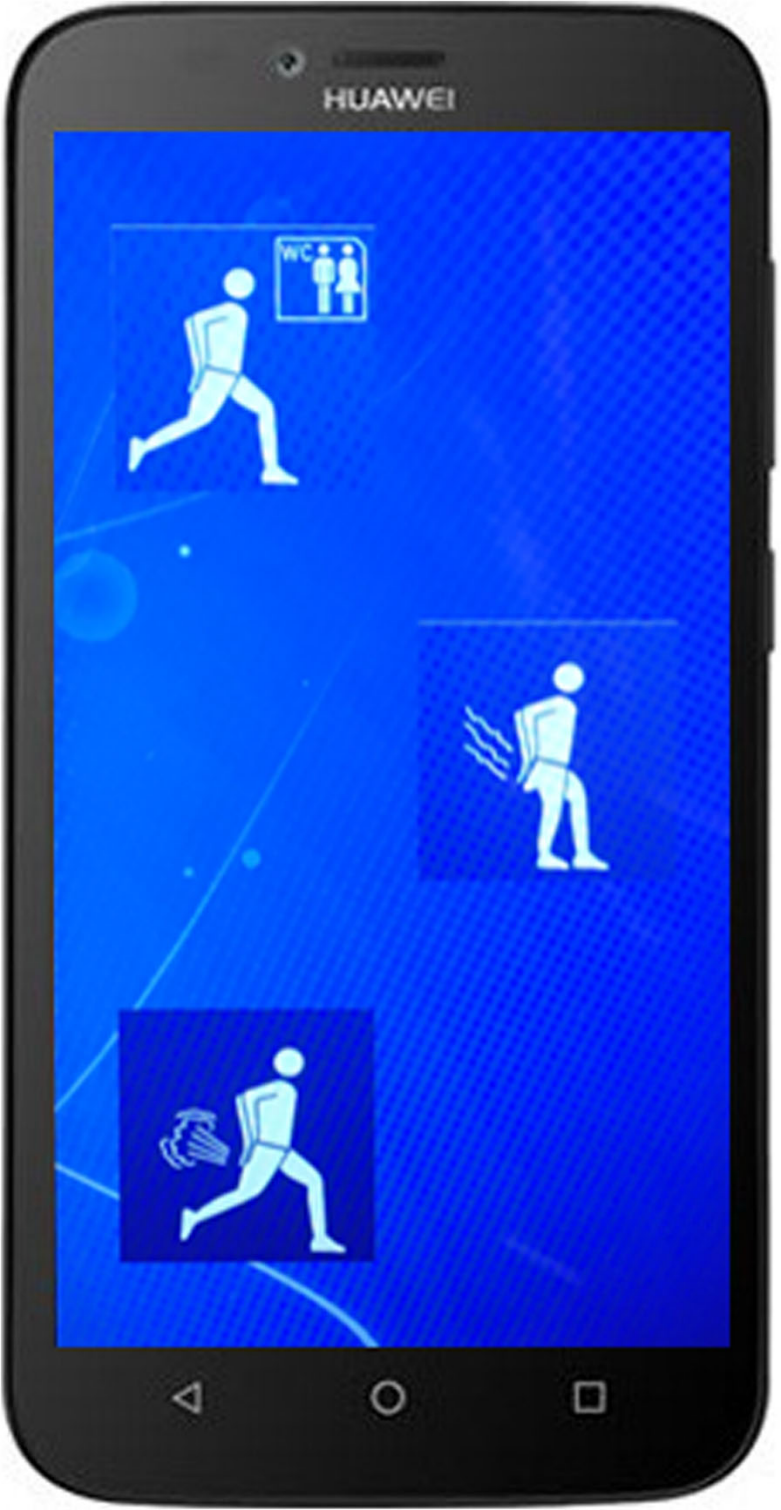
Example photograph of touch-screen icons on e-recording device

A variety of quality of life questionnaire and bowel diary measures recorded at 16, 32 and 58 weeks:

1. E-event recorder including episodes of faecal material, leakage of flatus, urgency without incontinence, social and physical activity (Fig. 5)
2. Other bowel diary measures: urgency, urge and passive faecal incontinence episodes, use of loperamide and social functioning
3. Summative questionnaire assessments: St Mark’s continence score [31]; OAB-Q SF score, FI QoL score [32]; International Consultation on Incontinence Bowel (SF-ICIQ-B) questionnaire [33]
4. Viscerosensory bowel diary recording quality, site and intensity of defaecatory urge
5. Generic QOL: EQ-5D-5 L
6. Likert scale of patient’s global impression of treatment success (scale 0–10) and patient perception of group allocation (blinding success)
7. Electrode settings (including motor, first and habituated sensory thresholds), programming, and, if applicable reprogramming data
8. Adverse events and morbidity

### Mechanistic outcomes

#### Anorectal sensorimotor function

1. Frequency of perceived and unperceived TASRs per unit time (pre- and post-prandial)
2. Anal sensory electrical threshold
3. Rectal volumetric thresholds (minimum, urge, maximum tolerated) to balloon distension

#### Anocortical function

Magnetoencephalography (MEG): recordings will be acquired in response to anal electrical stimulation at 75% pain threshold, voluntary anal squeeze, and to acute supra-sensory sacral-root stimulation. A synchronous anal electromyogram (EMG) will also be recorded to aid interpretation and a control area utilised (median nerve). Whole cortical data will be obtained using standard methods on an Elekta Triux 306 channel system utilising noise cancellation methods to eliminate implant and stimulator artefacts. A beam-former analysis methodology will be employed to evaluate both evoked and induced changes in brain activity associated with SNM and anal stimulation. Brain sources will be constructed using individual co-registered T1-weighted MRI brain volumes. The outcome of this process will be a measure of the changes in brain oscillatory power and/or frequency changes computed from brain structures where maximum changes associated with anal stimulation are observed. These changes will be depicted in statistical brain volumetric images.

### Statistical considerations

#### Sample size

The sample size is based on the primary outcome; i.e. faecal incontinence episodes per unit time as recorded using the 4-week bowel diary at the end of each 16-week crossover phase.

We assume that when the device is inactive, the average number of events in 4 weeks for a typical participant is 28. The number of events per month for that individual will have an over-dispersed Poisson distribution, with 95% range 7 to 112. But individuals will also vary, so the average number of events in a month could vary from 14 in an individual at one extreme to 56 in an individual at the other. This means that the correlation between log(number of events) for the same individual in two different months will be 0.2, and the standard deviation of log(number of events) for each month will be 0.775 (this is consistent with results from two previous NIHR trials in similar populations [18, 34], and with our clinical experience). Thus, to detect a 30% reduction in FI event rate with 90% power at the 5% significance level with a crossover design requires 80 participants. Allowing for 10% loss to follow-up a total of 90 participants will be randomised. This sample size would also achieve more than 90% power to detect a 50% reduction in FI event rate using the data from the first period of the crossover alone. This sample is also sufficient to detect changes in mechanistic outcomes (90% power) based on pilot data; i.e. using a one-sample test comparing logarithm of anal electrical sensitivity post-SNM, the proposed mechanistic sample size *n* = 25 will be sufficient to detect a 30% reduction in sensory threshold with 95% power at the 5% significance level, assuming the standard deviation of the change in log-sensitivity is 0.47 (consistent with a coefficient of variation of 0.5 for sensitivity, as observed in pilot data, and a correlation of 0.5 between pre- and post-SNM assessments). The anocortical studies are mainly exploratory and sample size will be based on success of recruitment. It is, however, envisaged that approximately 15 patients will complete all three visits for MRI/MEG. Previous MEG studies have drawn important conclusions with sample sizes of this order.

#### Method of analysis

##### Efficacy: primary analysis from crossover study

This analysis will be completed by the statisticians at PCTU.

The analysis of the primary outcome will compare SHAM and active therapy in both arms of the crossover trial, at T12–T16 and T28–32, using mixed Poisson regression analysis to adjust for a fixed effect of period and a random effect of individual. To allow observed numbers of events before and after activation in the same individual to have an over-dispersed Poisson distribution we will also include a random effect of time within individual. We will analyse all non-missing data, adjusting for the stratification variables (random effect of centre and fixed effect of sex). This approach is unbiased if missingness is related to observed outcome data or stratification factors from the same participant (a ‘missing at random’ assumption): further sensitivity analyses will explore this assumption if needed.

Secondary outcomes will be analysed in the same way – using Poisson regression for outcomes that are counts, and linear regression for other quantitative outcomes.

Exploratory analyses may also be performed using geospatial data from the touch-screen devices to calculate, e.g. number of outings from primary residence, as well as distance travelled and velocity (a surrogate for mode of transport), and to produce numerical and graphical summaries aggregated by trial arm.

##### Efficacy secondary analysis from cohort study

As in the primary efficacy analysis, mixed Poisson regression will be used to compare the primary outcome at T52–58 with baseline in all randomised participants, adjusting for a random effect of individual, and a random effect of time within individual (over-dispersion).

#### Mechanism studies

Data from the subset of patients undergoing advanced anorectal studies (*n* = 25 approx) will be collected during each phase of the crossover. These data take the form of counts, e.g. number of events, and continuous measures such as pressure. Data will be analysed as for secondary outcomes in the efficacy analysis.

Anocortical studies will be analysed by the Aston Brain institute using existing bespoke computer analysis packages (Graph (Elekta TM); Matlab TM and FieldTrip TM and SPM8 TM). A beam-former analysis methodology [35] will be employed to evaluate both evoked and induced changes in brain activity associated with SNM and anal stimulation. Group analysis of this data will allow determination of cortical reorganisational changes associated with chronic SNM. This will be achieved by the spatial normalisation of individual MRI volumes into a grid based on the Montreal Neurologic Institute (MNI) standard template. Statistical analysis will employ a non-parametric cluster-based permutation test^57^. Firstly, an uncorrected dependent-samples *t* test will be performed on pre- and post-stimulus brain activity across the entire brain volume. All voxels exceeding a 5% significance threshold will be grouped into clusters. A null distribution will be obtained by randomising the condition label (pre- or post-stimulus data) 1000 times and calculating the largest cluster-level *t* value for each permutation. This methodology has been shown to adequately control for issues of multiple comparisons.

### Confidentiality

Information related to participants will be kept confidential and managed in accordance with the Data Protection Act 1998, The Data Protection Directive 95/46/EC, NHS Caldecott Principles, The Research Governance Framework for Health and Social Care, ICH Good Clinical Practice Guidelines (1996) and the conditions of Research Ethics Committee Approval and current local regulatory requirements.

All CRFs will be pseudonymised. The participant’s will consent to their general practitioner (GP) and or referring clinician to be informed of their participation in the study.

### Access to data and archiving

The trial data will be made available to suitably qualified members of the research team, study monitors and auditors, the REC and regulatory authorities as far as required by law. When the research trial is complete, it is a requirement of the Research Governance Framework and Sponsor Policy that the records are kept for a minimum period of 20 years (as per sponsor requirements).

### Adverse events (AEs)

All AEs will be recorded on the CRF and in the medical notes. Severity, causality (relationship to study procedures) and assessment of seriousness will be at the discretion of the medically qualified individual (e.g. principal investigator or delegated member of team).

### Expected events

Expected AEs include

- Bleeding
- Pain
- Wound infection
- Worsening of, or de novo urinary incontinence
- Worsening faecal incontinence
- Unwanted/undesirable stimulation effects
- Numbness at neurotransmitter site
- Technical device issues including lead migration and fracture

Expected serious adverse events (SAEs) are those related to routine use of SNM. These are:

- Infection of lead or implantable pulse generator (IPG) necessitating removal or admission for intravenously administered antibiotics
- Unwanted stimulation effects necessitating device removal
- Lack/loss of efficacy necessitating device removal
- Revision of IPG placement due to discomfort or displacement
- Revision or removal of IPG due to technical device failure (including fractured lead or failure of impedance check on all four leads)
- Unrelated hospitalisation; e.g. elective surgical procedures or injury or acute medical problems

### Notification and reporting of SAEs

SAEs that are considered to be ‘related’ and ‘unexpected’ are to be reported to the sponsor within 24 h of learning of the event and to the Main REC within 15 days in line with the required timeframe.

### Monitoring and auditing

The PCTU quality assurance manager will conduct a study risk assessment in collaboration with the CI. Based on the risk assessment, an appropriate study monitoring and auditing plan will be produced according to PCTU SOPs. This monitoring plan will be discussed and authorised by the sponsor before implementation. Any changes to the monitoring plan must be agreed by the PCTU quality assurance manager and the sponsor. Audits may be conducted by the sponsor or funder representative. The study may be identified for audit via the risk assessment process, investigator or department request, allegation of research misconduct or fraud or a suspected breach of regulations or selected at random.

### Trial committees

The project will be under the auspices of the chief investigator and the PCTU. The project will be overseen by a Trial Steering Committee (TSC). The role of the TSC is to provide overall supervision of the study on behalf of the sponsor and funder to ensure the study is conducted in accordance with the principles of Good Clinical Practice (GCP) and relevant regulations.

The responsibilities of the TSC will include: ensuring that views of users and carers are taken into consideration; advising on the trial protocol; advising on changes in the protocol based on considerations of feasibility and practicability; assist in resolving problems brought to it by the Trial Management Group (TMG); monitor the progress of the trial and adherence to protocol and milestones; consider new information of relevance from other sources; consider and act on the recommendations of the Data Monitoring and Ethics Committee (DMEC), sponsor and/or REC; review trial reports and papers for publication.

A Trial Management Group (TMG) will meet monthly initially during study set-up and then less frequently, every 2 months. The TMG will be responsible for day-to-day project delivery across participating centres, and will report to the TSC.

A Data Monitoring and Ethics Committee (DMEC) will be convened. The DMEC will meet at least 4 weeks prior to the TSC to enable recommendations to be fed forward. The DMEC will review unblinded comparative data, monitor these data and make recommendations to the TSC on whether there are any ethical or safety reasons why the trial should not continue. The DMEC membership will be in accordance with NIHR/MRC as well as PCTU guidelines.

### Risks / benefits

#### Efficacy study-related risks

The study poses no major risk to participants above the standard risk of SNM therapy. SNM is an established therapy whose main attraction is non-invasiveness and safety compared to other surgical procedures. The short period (3 months) without active therapy imposed by the crossover design is not deemed ‘harmful’ for a chronic and stable condition by the time that surgical intervention is considered. The main procedural risks are unwanted stimulation effects: muscle spasms, vaginal pain, scrotal pain, leg pain and paraesthesia (common to some degree but manageable usually by reprogramming), infection (cited at 2%) and leading to device erosion or removal. Other listed AEs (based on FDA: PMA P080025) include: unwanted changes in bladder function (urgency, retention); pain at neurostimulator and/or lead site including skin irritation; and allergic or immune system response to the implanted materials that could result in device rejections. Malfunction of the components of the InterStim Therapy System including neurostimulator programming error, lead migration/dislodgement, lead fracture, erosion of the lead into the colon with perforation, neurostimulator battery depletion, extension fracture, neurostimulator migration can also occur.

Taking the average natural background radiation in the UK to be 2.3 mSv per annum, then an effective dose of 1.6 mSv for this study is approximately equal to 8 months of natural background radiation exposure. X-ray examination involves exposure to ionising radiation and carries a risk of induction of excess cancers which may not be expressed for many years after exposure. Using the adult population lifetime risk coefficient of 5% per Sievert gives a lifetime risk of cancer of approximately 1 in 12,500. The Public Health England Radiation Protection Division describes risks of this magnitude as very low.

Some of the questionnaires contain personal questions about bowel problems and the effect of these on quality of life and psycho-behavioural functioning; however, all have been used in studies of similar patients previously.

#### Mechanistic study related risks

For anocortical tests, the patient must be able to submit for a pre-study registration MRI, have a plug anal electrode inserted and sit in the MEG scanner for a total of about 45 min; the patient must attend three times. These tests are non-invasive and only confer mild discomfort due to insertion of anal catheter. No ionising radiation is employed by any tests.

For the anorectal tests, the main difference from routine clinical evaluation of anorectal function is the addition of prolonged high-resolution anorectal manometry. This test is not performed routinely and has a longer duration than standard studies (about 110 min); the patient must attend twice. These tests are non-invasive and only confer mild discomfort due to insertion of anal catheter. No ionising radiation is employed by any tests.

#### Study benefits

Participants will receive a high standard of surgery using the latest technical optimisation and monitored care as consequence of the protocol. All participants will receive SNM therapy due to the crossover design. Participation will add to the knowledge base for determining the pathophysiology of disease and treating adults with FI.

### Dissemination

Scientific findings will be subjected to international reporting and peer review. We will direct this information to the following groups: study participants and carers who have been involved in the trial; charity links and patient groups; NIHR collaboration.

## Discussion

### Double-blind efficacy study

A double-blind, randomised crossover design is appropriate to experimentally assess clinical effect size and to study mechanism. The crossover will compare sub-sensory chronic sacral-root stimulation against SHAM stimulation with the well acknowledged advantage of statistical efficiency. It is, however, also acknowledged that such a study *must* improve on the previous four attempts at crossover studies to provide useful efficacy data. The proposed design will address the main criticisms of previous studies:

1. Adequate intervention periods to adequately assess response. We will have two 16-week periods (SNM and SHAM) in comparison with previous studies (maximum 1 month [15])
2. Adequate washout period and reduced risk of carryover effects. While the duration of carry over effects of SNM is unknown, the current study design allows for almost 3 months’ washout before outcomes are assessed, compared to a maximum in previous studies of 1 week [15]. Clinical experience suggests that this duration is adequate but we will, nevertheless, continuously monitor the kinetics of therapy and washout throughout the study using the newly developed e-recording tools
3. Adequate statistical power, we propose a completed crossover of 80 patients compared to previous maximum of 24 [15]
4. Reduced selection bias. Although the crossover design does not permit full adherence to an ITT principle; i.e. from start of trial therapy with test stimulation, we will randomise all newly implanted patients rather than patients who have already been selected on the basis of successful chronic therapy. Selected patients will thus be naïve to chronic stimulation and *all* consenting implanted patients will be randomised
5. Reduced attrition bias. We will continue assessments on all participants provided that the patient has not withdrawn consent. Patients who become unblinded to intervention would not, however, contribute data to analysis
6. Improved patient blinding. We will use the experience gained from the Durham-based NIHR RfPB TiLTS-CC study to maintain blinding
7. Improved assessment methods (e.g. diaries are collected for a longer period. As well as a paper diary an electronic simple touch-screen device will also be trialled)

We do, however, accept that the choice of design has some limitations:

1. Although it is acknowledged that a small proportion of patients prefer supra-sensory stimulation (about 10% in our clinical practice), especially in the short term, for double-blinding it is clearly necessary to mandate sub-sensory stimulation and we acknowledge that this is in effect an experimental variant of the therapy used in ‘real life’. We will, however, comply with routine clinical practice by having a reprogramming session at 6 weeks in each arm regardless of intervention status; i.e. in the SHAM arm this will be a ‘pseudo-reprogramming’ event. For the FI indication, a recent randomised observer-blinded comparison showed no difference in effects of supra- and sub-sensory stimulation [27] building on a small study that showed that therapeutic response threshold was significantly lower than sensitivity threshold [36]. However, we acknowledge that some differences in physiological results have been recorded for sub- and supra-sensory stimulation in the patients with slow-transit constipation [37] and this is acknowledged in the study title
2. We acknowledge that it is difficult (and labour intensive) to blind patients to SNM in crossover designs, particularly for patients who receive the intervention first and then have it switched to OFF. This proved a problem in a recent study of irritable bowel syndrome [38] in which 75% patients correctly identified that the stimulator was ON or OFF across all crossover phases. This noted, previous crossover studies of FI (accepting limitations in published documentation) have successfully blinded participants. This remains a risk for any placebo-controlled intervention where the number needed to treat is relatively small; i.e. the majority of patients can identify their stimulation status by the effect it has on their symptoms. This noted, the effect size of SNM vs. SHAM remains uncertain (a reason for performing the study)
3. The study does not address the long-term clinical effectiveness, cost-effectiveness and safety of SNM

### Cohort follow-up study

The primary study (double-blind crossover) will provide a robust estimate of experimental efficacy. The cohort of thus recruited patients, however, also provide an opportunity to study the outcome at a later time point with patient-decisive stimulation (sub- or supra-sensory) as would be normal for routine clinical practice. On this basis, patients will be followed up for a further 6 months to a total, therefore, of just over 1 year post implant (2 × 16 + 26 = 58) and outcomes recorded between 54 and 58 weeks (± 1 week). Such data will provide the first estimate of the outcome of optimised (internationally standardised) lead placement [25, 26] in adults with FI and also do so with the scientific rigor mandated by a prospective randomised study managed by a CTU (even if the intervention by this stage is ‘open label’). It is acknowledged that patients will only have actually had 36 weeks of stimulation (continuous or discontinuous depending on crossover sequence). However, published data indicate that outcomes at 6 months are almost identical to those at later time-points (accepting data censorship in some cohort studies).

### Trial registration

The trial is registered on a publically accessible registry: ISRCTN98760715 (Registered on 25 September 2017); http://www.isrctn.com/ISRCTN98760715.

### Trial status

The trial commenced recruitment in October 2017 and will take 18 months to recruit 90 patients. Recruitment milestones will be closely monitored.

## Additional file

Additional file 1: Standard Protocol Items: Recommendations for Interventional Trials (SPIRIT) 2013 Checklist: Recommended items to address in a clinical trial protocol and related documents. (DOC 115 kb)

## Abbreviations

AE: Adverse event
CI: Chief investigator
DMEC: Data Monitoring and Ethics Committee
EQ-5D-5 L: EuroQol Health Outcome Measure
FI: Faecal incontinence
FI QoL: Faecal Incontinence Quality of Life
GP: General practitioner
HRA: Health Research Authority
MEG: Magnetoencephalography
MRI: Magnetic resonance imaging
NICE: The National Institute for Clinical Excellence
OAB Q: Assessment of OverActive Bladder Symptoms
PCTU: Pragmatic Clinical Trials Unit
QoL: Quality of life
R&D: Research & Development
RCT: Randomised controlled trial
REC: Research Ethics Committee
SAE: Serious adverse event
SF-ICQ-B: International Consultation on Incontinence Bowel Questionnaire
SNM: Sacral neuromodulation
SOP: Standard operating procedure
TMG: Trial Management Group
TSAR: Transient Anal Sphincter Relaxations
TSC: Trial Steering Committee
